# Clinical and microbiota alterations in performance horses undergoing long-distance transport

**DOI:** 10.1093/jvimsj/aalag137

**Published:** 2026-07-13

**Authors:** Ananya Mahalingam-Dhingra, Daniela Bedenice, Debora Regina Romualdo da Silva, Melissa Mazan, Tiffany Hall, Wade Tenney, Giovanni Widmer

**Affiliations:** Department of Large Animal Clinical Sciences, Cummings School of Veterinary Medicine at Tufts University, North Grafton, MA 01536, United States; Department of Large Animal Clinical Sciences, Cummings School of Veterinary Medicine at Tufts University, North Grafton, MA 01536, United States; Department of Infectious Disease and Global Health, Cummings School of Veterinary Medicine at Tufts University, North Grafton, MA 01536, United States; Department of Large Animal Clinical Sciences, Cummings School of Veterinary Medicine at Tufts University, North Grafton, MA 01536, United States; Hagyard Medical Institute, Lexington, KY 40511, United States; Department of Large Animal Clinical Sciences, Cummings School of Veterinary Medicine at Tufts University, North Grafton, MA 01536, United States; Department of Infectious Disease and Global Health, Cummings School of Veterinary Medicine at Tufts University, North Grafton, MA 01536, United States

**Keywords:** equine pneumonia, respiratory microbiota, shipping fever, transport-associated disease

## Abstract

**Background:**

Transport-associated pneumonia contributes substantially to morbidity, impaired welfare, and economic loss in high-performance horses and may be associated with alterations in the respiratory microbiota.

**Hypothesis/Objectives:**

Examine the effect of long-distance transport on the respiratory microbiota and correlate changes in microbiota diversity and composition with systemic and airway inflammation.

**Animals:**

Seventeen client-owned performance horses transported from New England to Florida under optimized trailering conditions, and 12 non-traveling horses.

**Methods:**

Physical examination, blood testing, nasopharyngeal wash, endoscopy, tracheal aspirates, and thoracic ultrasonography were performed 48 h before, and 24 and 72 h after transport (T1-T3). Upper and lower respiratory microbiota were characterized using high-throughput 16S rRNA sequencing and correlated with clinical variables using constrained ordination. Transport effects were evaluated using repeated measures analysis of variance (ANOVA).

**Results:**

Cortisol concentrations decreased post-transport (*P* = .02), whereas ultrasound scores (*P* = .01) and serum amyloid A concentrations (*P* = .03) increased from T1 to T3. Timepoint explained a small but significant portion of microbiota variability (*P* < .001). Upper and lower airway microbiota differed, with the lower airway showing more β diversity (lower stability; *P* < .001). Together, timepoint and ultrasound scores explained 19% and 10%, respectively, of nasal and tracheal bacterial microbiota variability. Operational taxonomic units with the highest fit to timepoint and ultrasound were enriched for plant-associated bacterial taxa, mainly Hyphomicrobiales.

**Conclusions and clinical importance:**

Even under standardized, optimized transport conditions, respiratory microbiota alterations occurred in healthy, athletic horses, correlating with ultrasonographic evidence of pulmonary inflammation. Inclusion of the fungal mycobiome may further improve our understanding of transport-associated respiratory disease.

## Introduction

Shipping fever, or post-travel pneumonia, is the most common cause of transport-associated illness in horses.^1^^,^^2^ Bronchopneumonia, even when treated, can rapidly progress to pleuropneumonia, where infection extends into the normally sterile pleural cavity,^3^ resulting in prolonged hospitalization, increased treatment cost, and delayed recovery. Survival rates for pleuropneumonia are estimated to be as low as 43%, and nearly half of survivors never regain full athletic performance.^4^ Because high-performance athletic horses represent the majority of transported horses, with the United States equine industry being valued at $177 billion ($37.3 billion from the competition industry alone^5^), the economic impact of this disease is marked.

The pathophysiology of shipping fever mimics that of other common transport-associated illnesses, including bovine (BRDC) and porcine (PRDC) respiratory disease complexes. Factors including crowding, viral exposure, decreased airway ventilation, and increased inhaled particulates all work to dampen innate immunity.^6^ Head elevation, a common practice when shipping, decreases the efficacy of natural mucociliary clearance and substantially increases the bacterial load and inflammation in the lower airways.^7^^,^^8^ Even with looser rope tying, however, significant lower airway inflammation occurs.^9^

Prevention of shipping fever remains challenging. Prophylactic antimicrobial use lacks strong evidence of efficacy, and it may disrupt beneficial commensal bacteria and promote antimicrobial resistance.^10^^,^^11^ Immunostimulants, such as low-dose interferon α and granulocyte colony-stimulating factor, are not proven to reliably decrease pathology.^12^^,^^13^ Thus, there is a critical need to develop alternative strategies to prevent disease in association with long-distance travel in performance horses.

The respiratory microbiome of other species has shown promise in expanding our understanding of lung immunity and consequently offers prophylactic treatment options for lower airway disease.^14^ Disruption of the respiratory microbiome (dysbiosis) has been linked to disease in multiple species. Dysbiosis has been implicated as a direct cause of pneumonia in people affected by human immunodeficiency virus,^15^ chronic obstructive pulmonary disease,^16^ and cystic fibrosis.^17^ Respiratory dysbiosis also has been established in BRDC and PRDC.^18^^,^^19^ Further investigation of the role of long distance transport on the equine respiratory microbiome is critical to better understand the pathogenesis of shipping fever. In turn, doing so may increase opportunities for evidence-based interventions to decrease disease incidence.

The objective of our study was to characterize the effect of long-distance transport on airway inflammation and microbial populations in performance horses*.* We hypothesized that long-distance travel would alter the microbiota composition and relative bacterial abundance, and that these changes would correlate with respiratory and systemic inflammation.

## Materials and methods

### Study population and transportation

**Figure 1:**
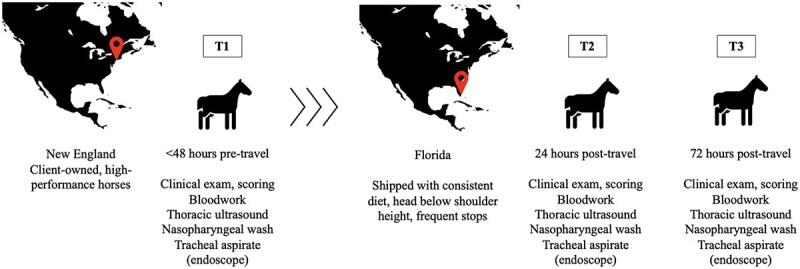
Study design. Horses were examined at 3 timepoints: T1 = 48 h before departure (New England), T2 = 24 h after arrival (Florida [FL]), T3 = 72 h after arrival (FL). Vital parameters, clinical scoring, hematology (CBC, inflammatory markers, cortisol), nasopharyngeal wash, endoscopy and tracheal aspirate, and thoracic ultrasound evaluation were obtained.

Our prospective field study enrolled 18 client-owned horses from a referral population in New England (NE) that were shipped to Florida (FL) in winter 2024 under standard guidelines. Horses were examined at 3 timepoints: T1 = 48 h before departure (NE), T2 = 24 h after arrival (FL), and T3 = 72 h after arrival (FL; Figure 1). Further details on horses, transport duration, and trailer dimensions are listed in Appendix A. Trailer particulate measurements were obtained during transport (Appendix A).

Exclusion criteria were signs of clinical respiratory disease or active inflammation (abnormality in more than 1 category for physical examination, serum amyloid A [SAA], CBC) or administration of anti-inflammatory or anti-microbial drugs during the study period. The experimental protocol was approved by the Tufts University Institutional Animal Care and Use Committee (#G2024-96) and completed after written client consent. Previously banked samples from non-traveling horses, taken 4 days apart using the same methodology, were used as stationary controls.^20^

### Clinical variables

Clinical examination, hematology, nasopharyngeal wash (NP), and endoscopic tracheal aspirates (TA) were performed as previously described.^20^ Thoracic ultrasonography was performed and later blindly scored based on an adapted scoring system^21^ (score 1-5) outlined in Table 1. All clinical variable methodologies are detailed in Appendix A.

**Table 1:** Scoring system used for thoracic ultrasonography.

**Pathology (per intercostal space)**	**Score**
**Normal pleural surface**	0
**1-5 comet tails**	1
**>5 comet tails**	2
**Mild consolidation (<2 cm)**	3
**Moderate consolidation (2-5 cm)**	4
**Severe consolidation (>5 cm)**	5

### DNA extraction and 16S PCR procedures

**Figure 2:**
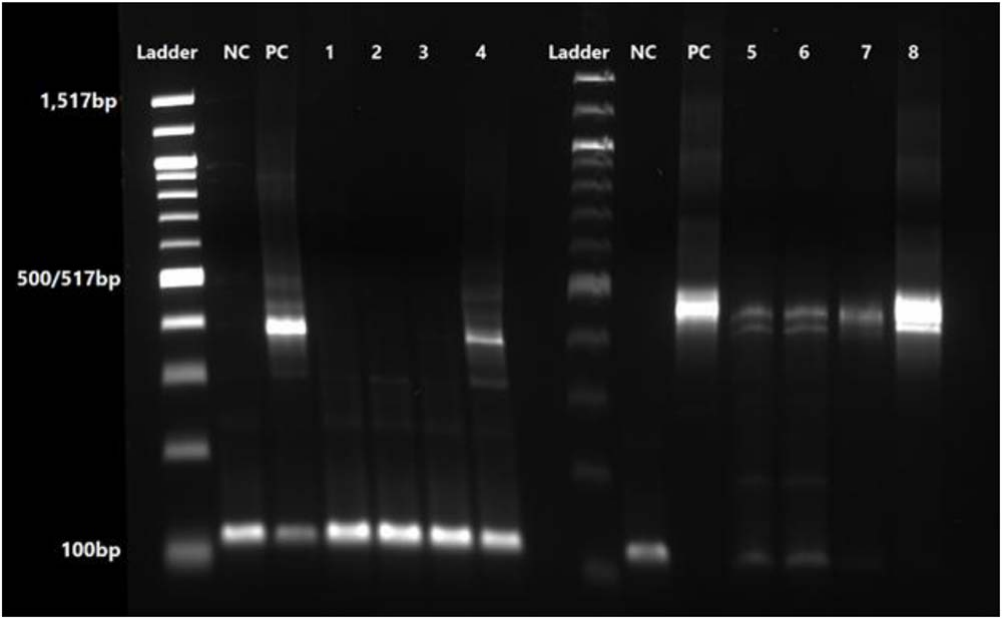
Agarose gel electrophoresis of 16S amplicons shows better amplification with V1V2 primers as compared with the more commonly used primers flanking the V4 variable region of the 16S rRNA gene. NC, no-DNA control. PC, positive control, DNA from a synthetic bacterial population (BEI resources, cat. no. HM-782D). Lanes 1–4, PCR with V4 primers; lanes 5-8, PCR with V1V2 primers. Lanes 1 and 5, sample 1 (tracheal aspirate); lanes 2 and 6, sample 2 (tracheal aspirate); lanes 3 and 7, sample 3 (TA); lanes 4 and 8, sample 4 (NP).

Previous PCR analyses of respiratory samples from healthy horses were hampered by relatively low PCR amplification rates.^20^ Different DNA extraction kits, temperature cycles, and annealing temperatures therefore were evaluated (outlined with library preparation in Appendix A). Final methodology utilized Qiagen DNeasy Blood and Tissue Kit followed by PCR amplification with primers specific for the V1V2 region of the bacterial 16S ribosomal RNA gene (Figure 2). The PCRs were run in a MIC Real Time thermal cycler (Biomolecular Systems, Potts Point, Australia).

### Statistics and bioinformatics

Clinical variables were tested for normality using the Shapiro–Wilk test and analyzed using repeated-measures analysis of variance (ANOVA) with post hoc analysis. Categorical variables (nasal cytology) were assessed using χ-square analyses. Statistical analyses were performed using SPSS (IBM SPSS Statistics, version 28.0. Armonk, NY: IBM Corp) and Sigmaplot version 15 (Systat, Santa Clara, California), with *P* < .05 considered significant. The significance of sample clustering according to sample type or trailer was tested with analysis of similarities (ANOSIM)^22^ as implemented in GenAlEx.^23^ Redundancy analysis (RDA) as implemented in CANOCO^24^ was used to assess the association between sampling timepoint, sample type, horse, ultrasound score, and respiratory microbiota. Further bioinformatics methodologies are listed in Appendix A.

## Results

### Horse population

Eighteen horses were recruited for the study and completed initial testing (T1). One horse did not travel to FL because of ownership changes, resulting in 17 horses completing the trip and being included in the final analysis after 3 days of testing (T1-T3). The first 12 horses (horses A-L) trailered from Medfield, Massachusetts to Morriston, FL, over the course of 2 separate trips (7 horses then 5; approximately 1300 miles). The second group of 5 horses (horses M-Q) was transported from Coventry, Connecticut to Ocala, FL (approximately 1200 miles). Of the 17 horses, there were 14 geldings and 3 mares. Breeds included Warmbloods (*n* = 11), Holsteiners (*n* = 3), Hanoverians (*n* = 2), and 1 Welsh Pony. Median [interquartile range] age was 11[6] years and mean (±SD) weight was 1238 ± 117 lb.

None of the 17 horses included in the study had any clinically relevant medical conditions at the time of enrollment. One horse had a previous history of a tie-back surgery (date unknown) before import as well as a history of hemosiderosis (without evidence of inflammatory airway disease) on bronchoalveolar lavage in July 2023. Two horses had a history of post-shipping pneumonia in previous years. None of the enrolled horses showed abnormal respiratory signs or systemic inflammation at T1. For the first trip of group 1 (horses A through G), the average particulate load in the trailer was 0.1007 mg/m^3^ and for the second trip (horses H through L) it was 0.0863 mg/m^3^. Particulate load during transport for horses in group 2 (M through Q) was 0.025 mg/m^3^.

### Effect of transport on clinical variables

**Figure 3:**
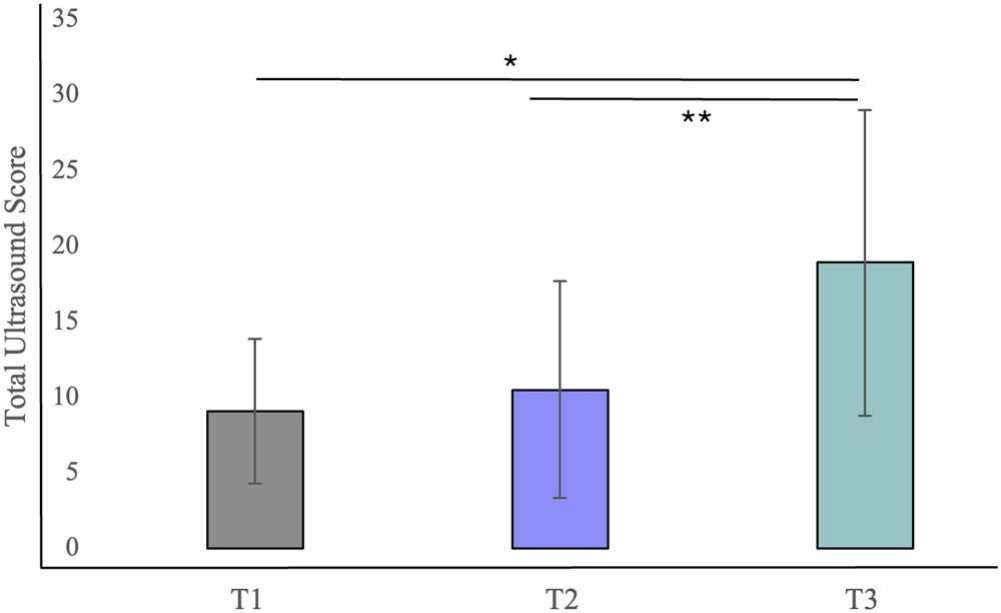
Total ultrasound score measured at different timepoints (shown as mean ± SD). Repeated measures analysis of variance with post hoc analysis showed significant difference between T1 and T3 (*P* = .01) and T2 and T3 (*P* = .01). Significance marked with * or **.

The following variables were analyzed using univariate analyses: respiratory rate, clinical score, SAA, mucus score, nasal cytology score, TA neutrophils, white blood cell count, neutrophil count, cortisol concentrations, pleural fluid, ultrasound score. A summary of these results is shown in Table 2. Respiratory rate (median [IQR], 12 [4] vs 16 [10] breaths per minute; *P* = .02) and SAA (median, 2 [10] vs 13.5 [138] μg/mL; *P* = .03) increased from T1 to T3. White blood cell count increased from T1 to T2 (6.3 ± 0.8 vs 7.56 ± 1.2 × 10^3^/μL, *P* = .002) and decreased from T2 to T3 (7.6 ± 1.2 vs 5.9 ± 0.9 × 10^3^/μL, *P* < .001). This tendency was mimicked by neutrophil counts, with an increase from T1 to T2 (3.6 ± 0.6 vs 4.8 ± 1.4 × 10^3^/μL, *P* = .01) and decrease from T2 to T3 (4.8 ± 1.4 vs 3.6 ± 0.7 × 10^3^/μL, *P* = .002). Ultrasound scores increased significantly from T1 to T3 (9.1 ± 4.8 vs 18.9 ± 10.1, *P* = .01) and T2 to T3 (10.5 ± 7.2 vs 18.9 ± 10.1, *P* = .01; Figure 3).

**Table 2:** Results of repeated measures analysis of variance (ANOVA) for clinical variables.

**Variable**	**Timepoint**	**Mean** ± **SD or Median [IQR]**	***P*-value (ANOVA)**
**Respiratory rate (breaths/min)**	1	*12 [4.0]*	.02
	2	*16 [8.0]*	
	3	*16 [10.0]*	
**Clinical score**	1	*1.0 [1.0]*	.08
	2	*1.0 [2.5]*	
	3	*1.0 [2.5]*	
**White blood cells (K/μL)**	1	6.3 ± 0.8 X 103/μL	.09
	2	7.6 ± 1.2	
	3	5.9 ± 0.9	
**Neutrophils (K/μL)**	1	3.6 ± 0.6	.01
	2	4.8 ± 1.4	
	3	3.6 ± 0.7	
**Serum amyloid A (μg/mL)**	1	*2 [10]*	.09
	2	*9 [14]*	
	3	*13.5 [138]*	
**Cortisol (μg/dL)**	1	3.1 ± 1.3	.02
	2	2.7 ± 1.0	
	3	3.0 ± 1.7	
**Mucus score**	1	*1.5 [2.1]*	.43
	2	*1.8 [1.6]*	
	3	*1.0 [1.6]*	
**Nasal cytology score**	1	*0.0 [0.0]*	.76
	2	*0.0 [0.0]*	
	3	*0.0 [0.0]*	
**Tracheal neutrophils (%)**	1	*4.0 [10.7]*	.40
	2	*39.3 [49.2]*	
	3	*21.6 [66.9]*	
**Pleural fluid average (mm)**	1	*2.7 [5.9]*	.37
	2	*1.6 [6.1]*	
	3	*3.3 [11.2]*	
**Ultrasound score**	1	9.1 ± 4.8	.09
	2	10.5 ± 7.2	
	3	18.9 ± 10.1	

Mean ± SD or median [interquartile range] (italicized) are reported, based on results of normality testing.

Three horses (M, K, and P) exhibited a disproportionally larger increase in markers of tracheal and systemic inflammation during transport (peak SAA 2257, 860, and 1992 μg/mL, respectively), compared with the remaining cohort. Tracheal neutrophil counts increased to >25% in each horse at T3 (range, 27.8%-85.6%), with visually apparent abnormalities in tracheal fluid color and opacity. Tracheal aspirate fluid cultures were obtained in 2 of these horses, based on concerns for development of bacterial infection, but yielded no growth. All 3 affected horses had a mild to moderate increase in total ultrasound score (range 1-9) over the course of the study period. However, different horses showed the largest overall increase in ultrasound score (range, 18-24) in the absence of notable increases of systemic or tracheal inflammation. Horses with parenchymal changes on thoracic ultrasonography or increased pleural fluid were followed up individually, and the observed abnormalities resolved over time without intervention.

### PCR optimization

DNA extracts were subjected to PCR amplification with primers specific for the V4 and the V1V2 regions of the bacterial 16S ribosomal RNA (rRNA) gene,^25^ with amplification of the V1V2 16S region deemed superior to the V4 variable region as assessed visually by gel electrophoresis (Figure 2). The optimal PCR conditions for amplifying the V1V2 region were as follows: denaturation for 1 m at 95 °C followed by 33 cycles of 95 °C for 5 s, 57 °C for 30 s and 72 °C for 20 s, with a final 5-m extension at 72 °C. The sequence of the 27F and 338R PCR primers flanking the V1V2 16S variable region were AGAGTTTGATCMTGGCTCAG and TGCTGCCTCCCGTAGGAGT, respectively. Previous studies using V4 primers resulted in an amplification rate of 57% and 14% for nasal and tracheal samples, respectively.^20^ With the V1V2 PCR amplification rates of 92% and 80%, respectively, were achieved.

### Effect of sample type on respiratory microbiota

**Figure 4:**
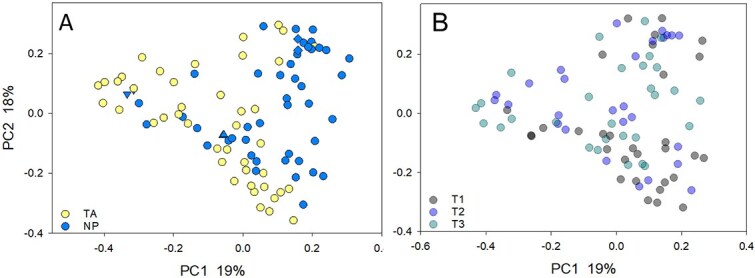
Principal coordinate analysis of respiratory samples colored according to sample type (A) and timepoint (B). The analyses are based on weighted UniFrac distance matrices. Triangle and diamond symbols in plot a show replicated samples. The percent variation explained by each axis is indicated. The clustering by sample type is statistically significant (ANOSIM *R* = 0.21; *P* < .001), but clustering by timepoint is not, according to the same test (*R* = 0.012, *P* = .22). Abbreviations: NP = nasopharyngeal wash; TA = tracheal aspirate; T1, T2, T3 = timepoints.

**Figure 5:**
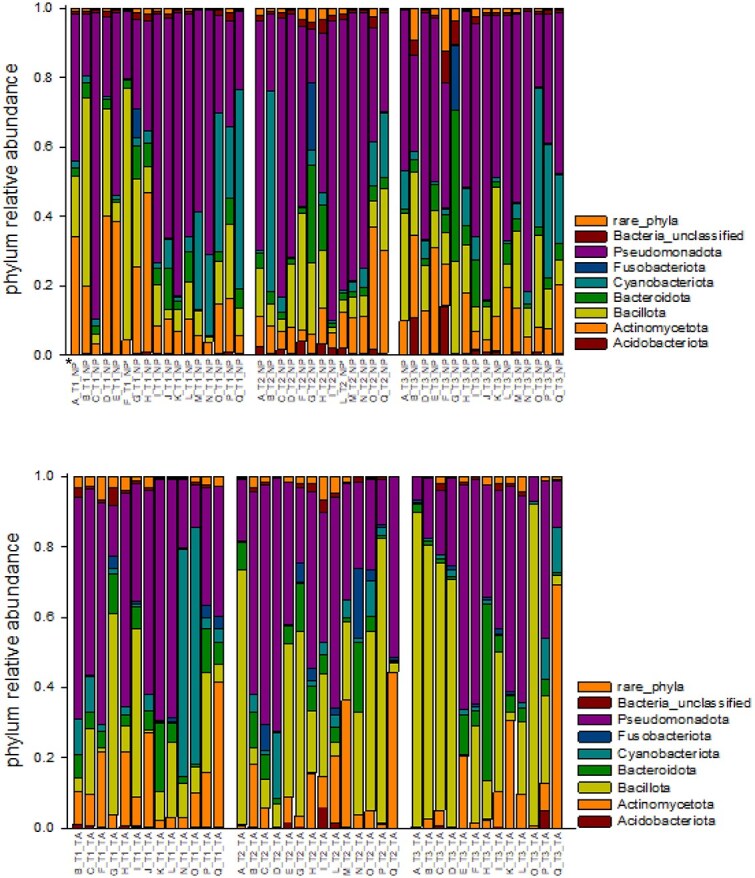
Phylum-level stacked bar graphs showing relative abundance of the 7 most abundant phyla in respiratory samples. Top, nasal wash pellet; bottom, tracheal aspirate. Within each plot, samples are grouped by timepoint (T1, left; T2, center; T3, right). Phyla with fewer than 0.5% of sequence reads are combined under “rare_phyla.” Asterisk (*) denotes mean of 2 independently sequenced amplicons from the same sample. The sample labels on the *x*-axis indicate horse_timepoint_sample-type.

DNA extracted from NP, TA, and endoscope samples (for evaluation of contamination) were analyzed using 16S PCR. Of the respiratory samples (NP and TA), 88/102 (86%) amplified. The proportion of NP samples that amplified (47/51, 92.2%) was slightly higher than for the TA samples (41/51; 80.4%). Only 1 endoscope sample amplified, and because of the low DNA concentration in this sample (11% of the corresponding tracheal sample), contamination of sequence data from the scope was likely insignificant. Sample type (NP vs TA) explained the largest share of operational taxonomic unit (OTU) variability (18.9%, pseudo-*F* = 19.8, *P* < .001; ANOSIM *R* = 0.21; *P* < .001; Figure 4). The taxonomic differences between NP and TA microbiota were identified using RDA defining sample type as independent categorical variable and the effect of horse, timepoint, and trailer (or property) included as co-variates. Specifically, we tabulated the taxonomic classification of OTUs that are best explained by the RDA model. Of the 20 OTUs with the best fit to sample type (>22% of OTU variation explained by the RDA model), 8 were in the class Bacilli, orders Staphylococcales and Lactobacillales. These taxa are listed in the “Best fit” column of Table 3. Of the 20 OTUs of similar relative abundance in NP and TA, with the lowest fit to the RDA model (<0.26% OTU variation explained), none belonged to the class Bacilli. In contrast, 10 of 20 OTUs in this low-fit group were classified as Hyphomicrobiales (Table 3), which are plant-associated bacteria.^26^ The association between fit (low or high) and taxonomy (Hyphomicrobiales or other) was significant (χ^2^ = 10.2, 1 df, *P* = .001). In most samples, Pseudomonadota (Proteobacteria) was the most abundant phylum regardless of sample type and timepoint. Phylum-level stacked bar graphs (Figure 5) show the relative abundance of the 7 most abundant phyla.

**Table 3:** Lowest taxonomic level classification of OTUs with best and worst fit to sample type and timepoint, respectively.

**Independent variable**	**Best fit** ^a^	**Worst fit** ^b^
**Sample type^c^**	Staphylococcus	Rhizobiaceae
	Aerosphaera	Marmoricola
	Streptococcus	Agrobacterium
	Micrococcaceae	Chloroplast_insertae_sed_genus
	Brachybacterium	Rhizobiaceae
	Gammaproteobacteria	Microbacteriaceae
	Pasteurellaceae	Beijerinckiaceae
	Streptococcus	Rhizobiaceae
	Rothia	Microbacteriaceae
	Pseudomonas	Microbacteriaceae
	Streptococcus	Actinobacillus
	Mammaliicoccus	Rhizobiaceae
	Corynebacterium	Bacillota
	Streptococcus	Devosia
	Staphylococcus	Rhizobiaceae
**Timepoint^d^**	Rhizobiaceae_unclassified	Sphingobium
	Rhodopseudomonas	Gammaproteobacteria
	Rhizobium	Devosia
	Sphingomonadaceae_unclassified	Neisseriaceae
	Methylobacterium	Acinetobacter
	Methylobacterium	Microbacterium
	Corynebacterium	Rhodococcus
	Geodermatophilaceae_unclassified	Sphingomonadaceae
	Rickettsiella	Simplicispira
	Beijerinckiaceae_unclassified	Rhizobiaceae
	Rhizobiaceae_unclassified	Microbacteriaceae
	Methylobacterium	Micrococcaceae
	Methylobacterium	Lysobacteraceae
	Roseomonas	Clavibacter
	Priestia	Microbacteriaceae

a Taxonomic classifications of OTUs with the best fit to the RDA model.

b Taxonomic classifications of OTUs with the worst fit to RDA model.

c Covariates: timepoint, horse, trailer set.

d Covariates: sample type, horse, trailer set.

**Figure 6:**
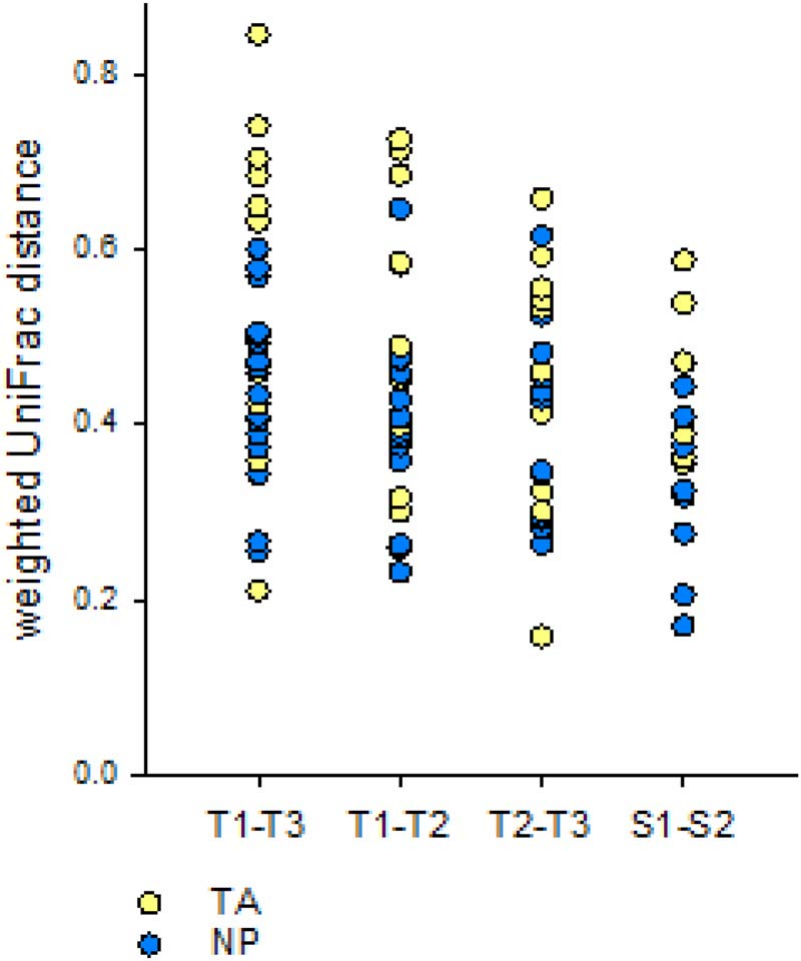
Comparison of respiratory microbiota temporal stability in shipped and stationary horses. Each datapoint represents 1 weighted UniFrac distance value between a pair of nasopharyngeal samples (NP, blue) or tracheal samples (TA, yellow) from the same horse. The β diversity values are stratified on the *x*-axis according to timepoint. T1, T2, T3 are from the present study; S1, S2 are from stationary horses^40^ and are used here as baseline. Average S1-S2 β diversity is smaller than T1-T3, consistent with transport driving microbiota divergence. The trend for higher β diversity between TA samples apparent on the *y*-axis indicates that TA samples are less stable than NP samples.

As shown in Figure 6, stratifying the β diversity values by sample type indicated that TA samples were less stable (larger β diversity) in all timepoint groups. Unique fraction metric (UniFrac) distances between TA samples from the same horse averaged 0.50 $\pm$ 0.16 (*n* = 40), whereas for NP samples the average was 0.39 $\pm$ 0.11 (*n* = 49). The difference was significant (*t* = −3.75, 87 df, *P* < .001). Nasopharyngeal wash and TA microbiota also differed with respect to evenness (*t* = −4.2, *P* < .001). This difference was apparent in a plot of rank-abundance curves (Figure S1) where TA rank-abundance curves tend to be flatter (ie, the TA bacterial populations are more even).

We were particularly interested in the abundance of sequences belonging to OTUs predicted to be symbiotic, as opposed to free-living, because of the expectation that many microorganisms in the upper respiratory tract, being inhaled, would be free-living. Contrary to this expectation, the abundance of sequences from predicted free-living bacteria was higher in TA microbiota as compared with NP. In a 2-way repeated measures ANOVA with sample type and timepoint as factors, the difference between TA and NP with respect to the abundance of predicted free-living bacteria was significant (*n* = 80, *F* = 9.90, 1 df, *P* = .01).

### Effect of transport on the respiratory microbiota

To understand the response of the equine respiratory microbiota to transport, timepoint was defined as an independent continuous variable ranging from 1 to 3 and analyzed using RDA. Sample type, horse, and trailer again were defined as co-variates. Timepoint explained a small (5.2%), but significant, portion of microbiota variability (pseudo-*F* = 3.7, *P* < .001). However, clustering by timepoint in the absence of co-variates was not significant according to ANOSIM (*R* = 0.012, *P* = .22). The effect of timepoint on the microbiota taxonomy inferred from the RDA showed environmental or plant-based bacteria in the class Alphaproteobacteria, genera Rhizobiales, Rhodopseudomonas, Methylobacterium, and Rosemonas to be negatively impacted by travel (Table 3). The association between abundance of Alphaproteobacteria and travel was significant (χ^2^ = 6.5, *P* = .01). Importantly, OTUs with abundance profiles that best fit timepoint tend to decrease in relative abundance over time (ie, these OTUs tend to be more abundant at T1 than T3). The only taxon positively associated with timepoint was genus *Priestia* in the class Bacilli, which increased over time.

We extended RDAs to include the cumulative ultrasound score together with timepoint as independent variables. Here, TA and NP samples were analyzed separately, defining trailer as co-variate and weighting OTUs according to the number of sequences in each OTU. The joint effect of timepoint and ultrasound score on the microbiota was significant (TA, *n* = 34, pseudo-*F* = 3.5, *P* = .004, 19.5% OTU variation explained; NP, *n* = 37, pseudo-*F* = 1.8, *P* = .04, 10.0% OTU variation explained). This analysis confirmed the positive correlation between timepoint and ultrasound we observed independently using Pearson correlation (*r* = 0.47, *P* = .002, *n* = 42). As observed in the global analysis comprising TA and NP samples described above, OTUs negatively correlating with timepoint and with ultrasound score were much more abundant than positively correlating OTUs.

### Effect of transport versus time on respiratory microbiota

To further explore the impact of travel on the respiratory microbiota, we evaluated the possibility that changes in the microbiota among T1, T2, and T3 were unrelated to travel, but resulted from changes in the respiratory microbial population over time. The same V1V2 PCR protocol was applied to generate 16S amplicons from TA and NP samples collected as part of a previously published study of stationary horses.^27^ The samples in this previous study were collected 4 days apart, before the horses were subjected to any treatment, and are designated here as S1 and S2. β diversity between S1 and S2 samples, quantified using the weighted UniFrac distance,^28^ was calculated between the same sample type and the same horse. For the current study of horses shipped to FL, β diversity values were grouped into the 3 categories T1-T2 (*n* = 24), T2-T3 (*n* = 23), and T1-T3 (*n* = 27). For the stationary horses,^20^ β diversity values (*n* = 15) were tabulated between timepoints S1 and S2. A 2-way ANOVA using time interval (S1-S2, T1-T2, T2-T3, T1-T3) and sample type (NP, TA) as factors and weighted UniFrac distance as dependent variable returned a significant effect for both factors (timepoint *F* = 2.91, 3 df, *P* = .04; sample type *F* = 15.53, 1. df, *P* < .001). Of the 6 pairwise comparisons between time intervals, only T1-T3 vs S1-S2 approached significance, where the mean β diversity between microbiota from shipped horses was 0.11 UniFrac units larger than the average β diversity of stationary horses (*P* = .05; Figure 6). These results are consistent with the RDA analysis described above in showing that transport had an impact on the respiratory microbiota beyond what can be expected in stationary horses.

## Discussion

Our study found that transport of healthy, athletic horses, even under optimized shipping conditions, induces significant changes in inflammatory markers and pulmonary parenchyma. Previous studies have established that transport induces systemic inflammation, most commonly indicated by leukocytosis and an increase in acute phase proteins.^29^^,^^30^ More recent studies examining restriction of head movement found similar induction of inflammation even with long-rope tying,^9^^,^^31^ in agreement with our findings. Although inflammatory markers changed in our study population, these concentrations remained within reference range for most horses. In contrast, a subset of 3 horses showed a disproportionate increase in markers of systemic and tracheal inflammation compared with the remaining cohort, indicating that these variables are most useful for the individual characterization of horses over time.

Previous studies have shown an increase in cortisol concentrations immediately post-transport, unlike the initial decrease in cortisol (T2 compared with T1) observed in our study. Although increased cortisol concentrations have been associated with stress during travel,^9^ alterations in cortisol also may be related to an inflammatory response. Inflammatory mediators released during disease decrease secretion of corticotropin-releasing hormone and ACTH, thereby decreasing cortisol production.^32^ Additionally, timing restrictions imposed by a client-owned study population may have contributed to cortisol variability, considering that basal cortisol secretion varies substantially with the circadian rhythm.

Although thoracic ultrasonography is limited in its ability to detect deep lesions distant from the pulmonary surface, it is an established imaging modality with moderate sensitivity and high specificity in horses for the diagnosis of bacterial pneumonia.^21^ Thoracic ultrasound is more available and considered superior to radiographs in detecting pulmonary changes, such as localized consolidation and pleural fluid, in adult horses.^33^ A prior study showed good conformity between thoracic ultrasound and post-mortem findings in foals with acute interstitial pneumonia, whereas the interpretation of thoracic radiographs was considered highly variable.^34^ Although thoracic imaging in adult horses cannot be directly compared with foals because of the depth of the chest cavity, evidence suggests that this imaging modality complements the clinical assessment of early airway inflammation, which cannot be determined by tracheal cytology alone.^35^^,^^36^ Our results not only established that long distance transport of healthy performance horses can induce pulmonary parenchymal pathology, but also that these changes correlate with alterations in respiratory microbiota during transport. More specifically, ultrasonographic lung changes correlated with decreased α diversity in respiratory samples after transport.

Our study confirmed significant differences in upper (NP) and lower (TA) respiratory microbiota, as previously observed in horses^27^^,^^37^ and other species. Studies in humans, cattle, and swine have characterized the populations and differences in upper respiratory compared with lower respiratory microbiota, and that alterations in both are associated with various lower airway diseases.^38–40^ Despite these established differences, it has been hypothesized that the lower respiratory tract microbiome, which is generally considered to be a lower biomass area, is heavily influenced by the upper respiratory microbiota. This situation is commonly known as the “adapted island model of lung immunity,” where the main contributor to the lower respiratory microbiome is influenced heavily by the entry and exit of microbes from the upper respiratory tract.^41^ It follows that in situations of increased particulate exposure, the nasal passage seeds oropharyngeal particulates into the lower respiratory tract.^7^^,^^8^ Particulate exposure for the horses in our study averaged 0.071 mg/m^3^, a level that has been correlated with lower airway inflammation.^42^ It was therefore expected that this degree of particulate exposure would result in a dynamic upper respiratory microbiome with consequent changes to the lower respiratory microbiome. Our results of relative evenness among NP microbiota samples and a more dynamic nature (greater β diversity) of TA samples are therefore unexpected. The observed high relative abundance of free-living bacteria in tracheal samples compared with nasal samples contradicts the assumption of stable commensal microbiota populating the trachea. Future studies examining the immune function of the lower respiratory tract may elucidate what roles inhalation and a specific immunologic signature play in shaping the respiratory microbiota.

Similar to our findings, taxonomic analysis of the equine microbiota in other studies have established Firmicutes (syn. Bacillota), Proteobacteria (syn. Pseudomonadota), and Actinobacteria (syn. Actinomycetota) as predominant phyla in the respiratory tract.^37^^,^^43^^,^^44^ Recent investigations of respiratory microbiota changes in association with the transport of research horses, observed an increase in Pasteurellaceae (Pseudomonadota) after travel, which was not found in our study.^9^^,^^31^ Our analysis showed that *Priestia* (Bacillus) was the only taxon to be positively associated with travel. Similarly, increases in the relative abundance of Bacillus sequences also were observed in a previous study.^31^ In general, OTUs that responded to travel decreased in relative abundance. Because of the compositional nature of the sequence data, it is conceivable that the relative abundance increases even though few bacteria are present in a sample. Most interestingly, our study found that some OTUs’ relative abundance decreased with timepoint and with increasing ultrasound score; Alphaproteobacteria, specifically Hyphomicrobiales, decreased with transport. Further classification determined that most were plant-based genera (Rhizobiales, Rhodopseudomonas, Methylobacterium). Given the association of these organisms with transport in our study, the prominence of plant-based organisms merits further investigation, including expansion of our scope to assess the respiratory mycobiome (fungal population).

Our study had several limitations. First, to control for the factor of time and to quantify the effect of transport on the microbiota, we used samples from stationary horses from a previous study.^20^ These samples were collected pre-intervention and only 4 days apart, which is not a direct comparison with the 6 days between T1 and T3 in the current study. Second, although 2 of the 3 groups of shipped horses came from the same barn and were transported in the same trailer but in 2 separate trips (A-G, H-L), the third group (M-Q) was separate, introducing variability, which was apparent in the sequence data. An additional potential contributor to the respiratory microbiome is the intestinal microbiome. All horses enrolled in the study remained on the same diet throughout the testing period to minimize potential effects unrelated to travel. However, the horses experienced variable levels of turnout in NE and FL because of the season, potentially impacting the respiratory microbiota. The impact of gastrointestinal changes on the respiratory microbiota merits exploration in future studies, because the gut–lung axis may provide more therapeutic options. Lastly, compared with other host-associated microbiota, particularly fecal microbiota, bacterial populations in the respiratory tract seem to be much less abundant. Numerous failed PCRs of respiratory samples, particularly bronchoalveolar lavage samples from a previous study in healthy horses,^20^ led us to explore the performance of primers other than the more commonly used V4 primers. Even though the V1V2 16S region is less frequently targeted, we opted for this protocol because of its higher PCR success rate. The drawback of this approach is that datasets obtained from different 16S variable regions are difficult to compare.

In summary, our study established that inflammatory, pulmonary, and microbiota alterations were associated with standardized, long-distance transport in a healthy population of athletic horses. Ultrasonographic evidence of pulmonary pathology was associated with respiratory dysbiosis. However, because of the limitations of working with client-owned horses, our study cannot establish whether the respiratory microbiota plays a substantial role in the development of shipping fever or if alterations in these bacterial populations respond to travel-related physiological and environmental changes. Future studies examining the mycobiome, the immune function of the lower respiratory tract, and the gut-lung axis may advance our understanding of transport-associated disease in horses.

## Supplementary Material

Supplementary_material_aalag137

## Off-label antimicrobial declaration

The authors declare no off-label use of antimicrobials.

## Institutional animal care and use committee or other approval declaration

Tufts University Institutional Animal Care and Use Committee protocol #G2024-96, University of Connecticut protocol A22-025.

## Appendix A

### Transportation

Stationary horses were sampled in Storrs, Connecticut (approximately 75 miles from barn 1 and 7 miles from barn 2). The first 12 horses (horses A-L) trailered from Medfield, Massachusetts, to Morriston, FL over the course of 2 separate trips. Horses were shipped in a 2017 Exiss, 6-horse, head-to-head trailer. Box stalls measured 91″ $\times$ 30″, 88″ $\times$30″, and the center stall 86″ $\times$ 90″, bedded with pine shavings. Stops were made every 3 h. The third group of 5 horses (horses M-Q) was transported from Coventry, Connecticut, to Ocala, FL. Horses were shipped in a Cimarron, 6-horse, head-to-head trailer, in box stalls (82″ $\times$ 39″, 84″ $\times$ 39″, center box stall 112″). Stalls were bedded with pine shavings, and stops were made every 3.5 h for at least 30 min. Horses had their head loose or tied below shoulder height.

Owners and trainers were instructed to keep horses on the same diet in NE, throughout the journey, and upon arrival in FL. Horses did not undergo additional transport during the study period. All horses were active performance animals on the hunter/jumper show circuit, and actively in work (ridden 5 or more times per week), and were routinely shipped over 5 times per year around NE, New York, and to FL. Selected horses received IV fluids with supplemental vitamin B or given omeprazole before travel. The bulk of their diet consisted of timothy and alfalfa hay with small volumes of supplementary grain (senior grain, rice brain, Tribute Kalm ‘N Eazy® pellets), which remained unchanged during transport. All horses were up to date on vaccines for common respiratory pathogens (equine influenza, strangles, herpesvirus) and had been dewormed within 6 months of shipping. Horses were rested for at least 48 h after arrival in FL.

#### Clinical examination

Clinical examinations by a veterinarian included careful thoracic auscultation and use of a previously validated but adapted 20-point respiratory scoring system.^45^ The latter is a combined assessment of respiratory rate, effort, and inflammation. Weight tape measurements and body condition score were obtained by a single veterinarian.

#### Hematology

Blood was obtained at each timepoint, before the start of any clinical procedures, and centrifuged at 1790 *g* for 15 min (repeated if necessary to ensure separation). Serum and plasma samples were immediately frozen on dry ice before transfer to a −80 °C freezer for further analyses. SAA was measured using a point-of-care assay (StableLab; Zoetis Inc) on whole blood. Whole blood (EDTA-stabilized) was submitted for CBC and plasma protein and fibrinogen concentrations (IDEXX laboratories). Serum samples were used to measure cortisol concentrations using immunoassay at the Cornell Animal Health Diagnostic Center.

#### Nasopharyngeal wash

Horses were sedated using a combination of detomidine (up to 0.01 mg/kg detomidine IV; Dormosedan 10 mg/mL, Zoetis Inc) and butorphanol (up to 0.01 mg/kg butorphanol IV; Torbugesic, Zoetis Inc). Personnel handling the equipment, samples and horses wore vinyl gloves that were changed in between procedures. A nasopharyngeal wash was performed as previously described.^20^ Samples were centrifuged using a tabletop centrifuge at 1790 *g* for 15 min. The pellet and 2-4 mL of associated supernatant were re-suspended and frozen on dry ice before transfer to a −80 °C freezer.

#### Tracheal aspirate

The nasal passages were cleaned and a tracheal aspirate (TA) performed using a disinfected 1-m endoscope (1800Endoscope.com LLC) as previously described,^20^ with a saline wash sample collected from the instrument for contaminant testing before each procedure. The TA sample was centrifuged using the same protocol as NP samples, and pellet and supernatant were individually frozen on dry ice before transfer to a −80 °C freezer.

Nasopharyngeal (NP) and TA samples collected and frozen in FL were shipped overnight on dry ice to Tufts University for further processing.

#### Cytology

Three slides of each wash sample (nasopharyngeal and tracheal) were prepared after sample centrifugation and stained with Wright-Giemsa and Toluidine Blue.

#### Mucus scoring

Video endoscopy was used to assess amounts of tracheal mucus as a marker for airway inflammation. Blinded videos were assessed by 2 veterinarians and scored from 0 to 5 as previously described.^46–48^

#### Thoracic ultrasound

A Samsung E70 EVO portable ultrasound machine with low frequency (4-5 mHz) curvilinear transducer was used. Horses were lightly sedated for the procedure (0.2-0.4 mg/kg xylazine IV; xylazine HCl, 100 mg/mL, AnaSed LA; VetOne, Boise, Idaho, USA) if necessary and the haircoat was wetted with alcohol. Ultrasound video clips of the left and right thorax were recorded in each intercostal space (T3-T16, dorsal to ventral plane). A score of 1-5 was assigned for the severity of pathology per intercostal space, with each area of pathology counted separately (Table 1). The highest score per intercostal space was used for analysis, and the scores were summed for a total score per timepoint. Pleural space was quantified in depth (mm) of pleural fluid. Scans were included only if >50% of intercostal spaces could be visualized sufficiently to provide accurate scoring.

#### Particulate measurements

A laser photometer (SidePak™ Personal Aerosol Monitor AM510) was placed in the trailer at the approximate height of the horse’s thoracic inlet. Air was sampled at a flow rate of 1.8 L/min using a built-in 2.5 μm impactor to measure particulates with known penetration into the equine lung.^49^ The particulate count over the first 10 h of the trip was quantified as a time-weighted average (mg/m^3^).

#### DNA extraction troubleshooting

The following DNA extraction kits were tested in a QIAcube instrument according to manufacturer’s instructions: QIAamp PowerFecal Pro DNA Kit—Stool/Gut DNA Extraction (Cat. no. 51804), High Pure PCR Template Preparation Kit (Roche, Ref. 11796828001), Norgen Biotek™ Sputum DNA Isolation Spin Column Kit (Slurry Format) (Norgen Biotek Corp.™, SKU: 46200), and Qiagen DNeasy Blood and Tissue Kit (Cat. no. 69504). In addition, we applied the protocol described previously, based on the DNeasy Blood and Tissue kit supplemented with mutanolysin (300 U/mL) and lysozyme (20 mg/mL).^43^ The latter enzymatic treatment also was tested in combination with the Norgen Biotek™ (Thorold, Ontario) Sputum DNA Isolation kit.

#### Library preparation

Dual-barcoded sequencing libraries were prepared as previously described.^50^ Amplicon concentration was measured in a Qubit fluorometer (Invitrogen, Carlsbad, CA). Up to 96 amplicons were combined at approximately equal molar ratio. The libraries were size-selected using a PippinHT instrument (Sage Science, Beverley, MA) and sequenced paired-end 300 cycles in a MiSeq instrument (Illumina, San Diego, CA). FASTQ-formatted sequences were deposited in National Center for Biotechnology Information Sequence Read Archive under bioproject number PRJNA1339000.

#### Bioinformatics

A published bioinformatics pipleline^51^ using commands from the microbial ecology software *mothur*^52^ was used to assemble read pairs, de-noise, compute weighted UniFrac distances^28^ and taxonomically classify the sequences. The Silva V 138 SSU rRNA database was used as reference to classify V1V2 sequence reads. Sequences from each sample were randomly sub-sampled to 25 000 using the *mothur sub.sample* command with *persample = T*. After de-noising, the entire dataset included 1 802 618 sequences of which 178 980 sequences were unique. For quality control, a V1V2 16S amplicon amplified from a synthetic bacterial population DNA (BEI Resources, Manassas, Virginia, cat. no. HM-782D) was sequenced in the same library. To control for technical variation, 6 samples were duplicated by generating 2 separate 16S amplicons, each uniquely barcoded. The weighted UniFrac distance values between replicates were 0.09, 0.05, 0.09, 0.16, 0.29, and 0.18. Sequences were classified against the Silva reference taxonomy (release 138.1)^53^ using the Wang classifier.^54^ A 75% cut-off threshold was applied. Analyses were based on shared files created in *mothur* with the *make.shared* command and matching matrices of independent variables. Operational taxonomic units were obtained based on a 97% sequence similarity threshold. Those OTUs with fewer than 1 read per sample in average were culled. Phenotypic differences between TA and NP microbiota also were investigated using METAGENassist.^55^

## Abbreviations

ANOSIM: analysis of similarities
UniFrac: unique fraction metric
ANOVA: analysis of variance
BRDC: bovine respiratory disease complex
CFU: colony forming units
FL: Florida
IQR: interquartile range
NE: New England
NP: nasopharyngeal wash
OTU: operational taxonomic unit
PRDC: porcine respiratory disease complex
SAA: serum amyloid A
TA: tracheal aspirate
