# Supplementary material for: Clinical and microbiota alterations in performance horses undergoing long-distance transport
Source: J Vet Intern Med. 2026 Jul 13;40(4):aalag137. doi: 10.1093/jvimsj/aalag137 (PMC13362966; doi:10.1093/jvimsj/aalag137)
Supplement: Supplementary_material_aalag137 [file supplementary_material_aalag137.zip › Supplemental Figure.Cap.docx]

Supplemental Figure 1. Rank-abundance analysis of equine respiratory microbiota. Each dot represents the relative abundance of one OTU in one sample. OTUs are ordered decrementally by relative abundance from left to right. The log of normalized abundance is plotted on the Y axis and rank on the X axis. The steeper the slope, the less even the microbiota and vice versa. Note a tendency for higher evenness of TA microbiota as compared to NP. For clarity, lines connecting OTUs from the same sample are not shown. The apparent alignment of datapoints at Y=0.003 is caused by the replacement of zero abundance values with 0.003 so they can be displayed on a log scale. Blue, nasal wash pellet (NP); yellow, tracheal aspirate (TA).
