# Supplementary figures and images for: Clinical and microbiota alterations in performance horses undergoing long-distance transport
Source: J Vet Intern Med. 2026 Jul 13;40(4):aalag137. doi: 10.1093/jvimsj/aalag137 (PMC13362966; doi:10.1093/jvimsj/aalag137)

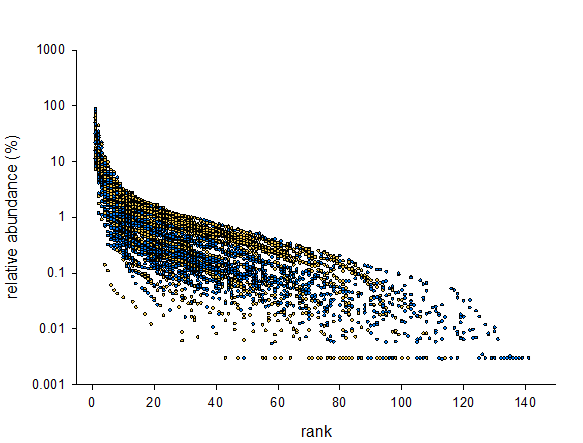

Supplement: Supplementary_material_aalag137 [file supplementary_material_aalag137.zip › Supplemental Figure 1.docx]
